# Methodological caveats in the environmental modelling and projections of climate niche for ticks, with examples for *Ixodes ricinus *(ixodidae)

**DOI:** 10.1186/1756-3305-7-S1-O18

**Published:** 2014-04-01

**Authors:** A Estrada-Peña

**Affiliations:** 1 Faculty of Veterinary Medicine. Zaragoza, Spain

## 

There is a growing interest in inferring the associations of health-threatening arthropods to capture the climate niche to which they associate, projecting such inference on a territory. This is intended to predict the range of distribution of the tick and to understand their responses to climate scenarios, using the so-called correlative models. However, some methodological gaps might prevent to obtain an adequate background against which test hypotheses. We explore, describe and illustrate these procedural inaccuracies with examples focused on the tick *Ixodes ricinus*, and how these may affect the modelling outcomes. We aim to provide a background of rules against which develop reliable models for these parasites. The use of partial sets of occurrences of the tick might produce unreliable associations with climate because the algorithms cannot capture the complete niche to which the tick is associated. Reliability measures of the model cannot detect these inaccuracies, and undesirable estimations of the niche will prevail in the chain of further calculations. The use of inadequate environmental variables (covariates) may lead to inflation of the results of the model through two statistical processes, called autocorrelation and colinearity. The high colinearity existing in climate products derived from interpolation of climate recording stations is demonstrated, and it is explicitly advised the training of climate models with satellite-derived information of climate, of which colinearity of the time series has been removed through a harmonic regression. The high uncertainty if inference on the climate niche is applied into different time slices, like projected climate scenarios is also pointed in the results.

